# Endorsement of Masculine-Typed Behaviors Decreases During Middle Adolescence: The Contextualizing Role of Peer Experiences for Adolescents Living in the United States

**DOI:** 10.1007/s10964-023-01861-z

**Published:** 2023-09-30

**Authors:** Jane Shawcroft, Adam A. Rogers, Matthew G. Nielson

**Affiliations:** 1 Department of Communication, University of California—Davis, Davis, CA USA; 2 School of Family Life, Brigham Young University, Provo, UT USA; 3 Department of Psychology, New York University—Abu Dhabi, Abu Dhabi, UAE

**Keywords:** Masculinity, Adolescent Gender Development, Accommodation, Resistance

## Abstract

While more research is emerging about the development of masculinity during adolescence, not much is known about how masculine-type behaviors develop over time in middle to late adolescence within the context of friendships and peer experiences. This study examined trajectories of masculine-typed behavior from ages 14 to 17. Multilevel modeling was used to account for cross-time and within-time variability in masculine-typed behavior and examined the role of positive and negative peer experiences in predicting this variability. This was done in a sample of 334 U.S. adolescents (51% boys; 50% White, 19% Black, 15% Latina/o/e). At the between-person level, boys and girls decreased in masculine-typed behavior over time. At the within-person level, negative peer experiences predicted fluctuations toward greater masculine-typed behavior, whereas friend support predicted fluctuations toward less masculine-typed behavior. Adolescence is a key period for navigating masculinity norms, and peer experiences are a key context for the development of masculine-typed behavior.

## Introduction

For individuals invested in supporting the healthy social and emotional development of adolescents, understanding the development of masculine-typed behaviors is paramount (Connell, 2005). Although masculine-typed behaviors – gender role norms of bravado and invulnerability – are often enacted within adolescent peer groups to gain status and belonging (Rogers et al., 2021), some masculine-typed behaviors, such as aggression, toughness, and emotional stoicism, are related to a range of psychological difficulties for boys and girls alike (Wong et al., 2017). Despite clear patterns of association between psychological difficulties and the accommodation of masculine-typed behaviors during adolescence (Wong et al., 2017), there are still several notable gaps in our understanding of processes of adoption of masculine-typed behaviors. These gaps significantly limit our ability to support healthy adolescent socio-emotional development. Specifically, little is known pertaining to the development of masculine-typed behaviors during later adolescence within the context of friendships, especially in samples including girls. Second, the cross-time trends that characterize the maintenance of this behavior during the second decade of life are also not well understood. This study aims to better understand how masculine-typed behavior develops during middle adolescence – particularly in the context of peer experiences. To gain insight into this core question, this study explores longitudinal patterns of change in adolescents’ behavioral conformity to masculine-typed behaviors during adolescence, specifically examining (a) intraindividual trajectories in masculine-typed behavior from ages 14 – 17, and (b) cross-time and occasion-specific predictors of masculine-typed behavior during this same period.

### Masculine-typed Behaviors in the Context of Adolescence

Scholarship has engaged in critical discourse about male gender-role norms and expectations across the lifespan, referred to collectively as “masculinity”. Researchers acknowledge that there are many “masculinities” within and across cultural contexts (Buschmeyer & Lengersdorf, 2016), with varying degrees of positive and negative meanings ascribed. Among these is a set of male role norms collectively referred to as *traditional masculinity*, which has been the subject of most empirical attention, being perhaps the most symbolically salient and recognizable construction of masculinity (Levant et al., 2011). Traditional masculinity is characterized as a bravado that outwardly projects invulnerability, manifested in behaviors like physical toughness, aggression when threatened, emotional stoicism, rugged individualism, status orientation, and aversion to the feminine (Levant et al., 2011).

Specific to adolescence, developmental scholars have established the relevance of traditional masculinity for informing the social lives of adolescents, particularly given that this is a period of intense identity formation (see Rogers et al., 2021, for a review). Changes associated with puberty usher in a heightened sensitivity for social belonging (Rapee et al., 2022), and gender-typed expression is an organizing criterion for whether adolescents find connection within their peer groups (Perry et al., 2019; Kleiser & Mayeux, 2021). As such, norms for traditional masculinity provide a familiar and recognizable script for developing a social identity and belonging in adolescence. For example, adolescents might be drawn to play aggressive sports (such as American Football or Rugby), make homophobic or sexist jokes among friends, or even avoid wearing “feminine” colors such as pink. Through such behaviors that broadcast traditional masculine traits – adolescents gain increased belonging to the wider peer group – which has been linked with increased well-being for adolescents (Roach, 2018).

Paradoxically, although traditionally masculine behaviors like toughness, individualism, stoicism, and aggression, can grant social status in the broader peer group (Jackson & Dempster, 2009), these behaviors concurrently undermine social and emotional competence and can lead to the attenuation of close social relationships (e.g., friendships; Way, 2011). Indeed, traditionally masculine-typed behaviors are associated with considerable psychological, social, and emotional difficulties for adolescents. Growing numbers of studies collectively evince the wide reach of these risks, linking masculine-typed behaviors to greater depressive symptoms (Rogers et al., 2017); relationship difficulties (Gupta et al., 2013), lower academic engagement and performance (Leaper et al., 2019); and risk behaviors such as alcohol use and abuse (Fugitt & Ham, 2018).

It is important to note that most of the literature on traditional masculinity has addressed boys’ and men’s engagement in masculine-typed behavior with relatively little consideration of girls’ and women’s own relations to such. The assumption frequently made is that masculine-typed behavior is most directly relevant to boys and men. However, girls occupy the same patriarchal contexts and institutions that devalue femininity (Chesney-Lind, 2011). In these contexts, girls report unique pressures related to adhering to traditionally masculine norms, often finding social utility in behaviors like emotional stoicism and toughness (Rogers et al., 2019). Indeed, girls may even perform masculine-typed behavior as a way of accommodating social systems that more highly value masculinity (Rogers et al., 2022). As such, this study included girls in its analysis to illuminate ways that girls both embody and perpetuate traditional masculine norms.

### Development of Adolescent Masculine-Typed Behaviors

Despite growing research on the relevance and implications of traditional masculinity in adolescence, very little is known about how adolescents come to uptake and maintain masculine-typed behaviors. Feminist frameworks are useful in guiding such predictions, having explored the ways that individuals navigate gender role norms within systems of power (Brown & Gilligan, 1992). Accordingly, individuals are not passive recipients of cultural gendered messages, but actively weigh the costs and benefits of conformity to gender roles for their social identities. Thus, individuals are engaged in ongoing negotiations, particularly in social and relational contexts, in which they conform to some gender role expectations – referred to as *accommodation* – while avoiding, downplaying, or actively opposing others – referred to as *resistance*. The classic demonstration of this was through work with adolescent girls, many of whom actively resisted feminine stereotypes for passivity (Brown & Gilligan, 1992). More recently, this theorizing has helped organize an understanding of boys’ negotiations of traditional masculinity. For example, empirical work on adolescent boys’ friendships (Way, 2011; Way et al., 2014) showed that nearly 80% of boys expressed concerns about the negative implications of traditional masculinity for their friendships, indicating that at least a desire to resist traditional masculine bravado is common in adolescence. That said, many boys in these studies still accommodated masculine norms to hedge against the risk of peer marginalization by appearing feminine, and this was particularly true in later adolescence. In sum, perceived social liabilities place adolescents in a balance of “resistance and accommodation” (Chu, 2014), in which they feel opposed to many masculine norms, especially within the context of friendship, but must still enact certain masculine-typed behaviors to ensure belonging within their larger social groups (Way et al., 2014).

#### Change in Masculine-Typed Behavior Over Time

One implication of the competing pulls for accommodation and resistance is that adolescents’ enactment of masculine-typed behavior is likely dynamic and in flux within and across time. Some studies have conceptualized this change in terms of developmental trajectories of masculine-typed behavior. These data show that traditionally masculine behaviors like physical toughness and emotional stoicism increase among boys of all observed racial/ethnic backgrounds as they enter middle school (early adolescence, or ages 11 to 14; e.g., Gupta et al., 2013). It is believed that during the transition to middle school, peer groups destabilize, and adolescents must navigate new, unfamiliar peer networks. In these novel peer contexts, boys may rely on familiar gender scripts (i.e., traditional masculinity) to establish a sense of belonging and social identity within their peer groups. This may explain boys’ greater uptake of masculine-typed behaviors. Less is known about girls’ trajectories of masculine-typed behaviors, though one study showed them to remain low and stable across this same period of development (Rogers et al., 2017).

Following early adolescence and the transition to high school, however, data become sparse, and trajectories of masculine-typed behavior in middle and late adolescence are unclear. As peer groups re-stabilize following the early adolescent period, some adolescents may settle into friendship groups and not feel as urgent a need to broadcast those same gendered traits and behaviors that secured for them a social status in earlier, more unstable peer arrangements. Furthermore, cognitive advancements in adolescence tend to afford greater flexibility (meaning an increased capacity to consider different perspectives, ideas, and attitudes) in how adolescents think about gender and gender-related behaviors over time. This increasing flexibility can lead adolescents to more pointed critiques of traditional gender roles, and therefore may translate into greater resistance to norms for masculine-typed behaviors. For example, one examination of trajectories of masculinity during later adolescence found young men to be, on average, less and less supportive in their cognitive endorsement of masculine-typed behaviors over time (Marcell et al., 2011). In addition, technological changes in how adolescents relate to their peers (such as social media) also offer opportunities for adolescents to see different perspectives on masculine behaviors.

Of course, conceptualizing change in terms of a developmental trajectory is only one way to examine variability in masculine-typed behaviors over time. The estimation of trajectories typically results in the interpretation of averages – including sample averages that reflect between-person differences in the rate of change (i.e., slope), as well as within-person averages that reflect a person’s own expected levels across repeated assessments (i.e., a person’s cross-time averages). While useful, these approaches do not always account for time-specific deviations that individuals experience from their own cross-time averages. Adolescents’ may fluctuate in their masculine-typed behavior beyond their own typical levels – (i.e., individual’s mean level of masculine-typed behavior across all measurement occasions). These fluctuations could provide meaningful information about developmental processes related to masculinity. For example, on occasions in which adolescents display more masculine-typed behavior than is typical for them, identifying factors that are reliably associated with those fluctuations can provide compelling insight in the what drives within-person developmental processes in masculinity. Therefore, this study examined change patterns in masculine-typed behavior during middle adolescence, accounting for cross-time (i.e., overall trajectories) and within-time (i.e., occasion-specific deviations from said trajectories) variability in these behaviors.

#### Predicting Change in Masculine-Typed Behavior

Feminist frameworks also emphasize the social embeddedness of gender role norms, including masculinity (Chu, 2014). That is to say, the enactment of gender roles, including masculine-typed behavior, happens primarily in the context of social and personal relationships (Rogers et al., 2021). Therefore, when considering factors that may reliably predict changes in masculinity across time and differences within measurement occasions, social and relational factors may be key. In adolescence especially, friends and peers are regular and salient social environments, and adolescents spend considerably more time with peers than with any other socializing agent (e.g., parents; teachers). Furthermore, status and belonging in many peer groups are directly tied to a adolescents’ ability to perform gender-typed behaviors (Perry et al., 2019), and it is common for adolescents to regulate gendered behaviors to promote group cohesion (Reigeluth & Addis, 2016). Given the centrality of peer and friend contexts in construing gender-roles, this study examined the role of positive and negative peer experiences in predicting cross-time and occasion-specific changes in masculine-typed behavior.

One commonly documented mechanism of gender-based “policing” is peer harassment. Peer harassment ranges from banter and teasing to outright victimization and ostracism. Recent longitudinal work has shown that boys and girls who are less typical of their gender group experience peer negative treatment at higher levels than gender-typical peers (Nielson et al., 2022). Gender-specific forms of harassment are also common policing mechanisms, especially homophobic name-calling (Reigeluth & Addis, 2016). For example, adolescents may use homophobic epithets to label certain traits as being “gay” or “feminine”, thereby learning to equate homosexuality and femininity with weakness and a loss of social power (Pascoe, 2014). This policing leads to the marginalization of less common social identities, while affording status to those who perform more traditional behavioral norms (Martin-Storey & August, 2016). Adolescents who experience more gender-based harassment may therefore try harder to accommodate masculine norms to avoid victimization, to gain a stable position in the peer group, or because masculine norms provide a familiar script for coping with social threats and rejection (Ioverno et al., 2021).

Conversely, more positive and uplifting peer interactions may also have implications for the performance of masculine-typed behavior. While some observations have noted that male-gender role socialization can undermine close interpersonal relationships (e.g., Gupta et al., 2013), the ability to maintain these close relationships may help adolescents resist masculine-typed pressures across time. Indeed, from a theoretical perspective, resistance to traditional masculine behaviors may stem from adolescents balancing their needs for social acceptance (often gained in the larger peer network via masculine behaviors; Kleiser & Mayeux, 2021), and the need for the support of close personal friendships. Indeed, close friendships satisfy critical attachment and intimacy needs for adolescents and can be an important source of solidarity and supportiveness (Brechwald & Prinstein, 2011). By having these belonging needs met, adolescents may feel a relative interpersonal security that can assuage felt pressures toward gender-role conformity as a means of fitting in. Indeed, studies suggest that adolescents who are more well-liked have more social “privilege” for resisting certain masculine norms (Way, 2011). In short, peer relationships and friendships are critical relational contexts that may give rise to traditional masculine behaviors, and as a result may predict variability in these behaviors across time.

## Current Study

Traditionally masculine norms represent a salient aspect of social landscapes in adolescence, and many adolescents experience competing pulls of resistance and accommodation to these norms. These realities have important implications for the uptake and maintenance of masculine-typed behaviors during adolescence. However, very little research has examined the patterns that characterize the development of masculine-typed behavior across time – especially during later adolescence and within the context of friendships. To better understand the development of masculine-typed behaviors over time within the context of peer experiences, the following analyses were completed. First, this study examined trajectories of masculine-typed behavior from ages 14 to 17 in sample of boys and girls from across the United States. The analysis examined intraindividual trajectories across time, while accounting for occasion-specific fluctuations in masculine-typed behavior around those trajectories. Given the relative sparsity of data to inform predictions as to the directionality of change, our research question here was exploratory. Second, this study examined the social and interpersonal antecedents of change in masculine-typed behavior. Indices of peer harassment (negative treatment, homophobic name-calling) and friend support were used as predictors of cross-time change (trajectories) and occasion-specific fluctuations in masculine-typed behavior. Based on theory and prior studies, it was hypothesized that experiences of peer harassment would predict change toward greater masculine-typed behavior, both across- and within-time. It was also hypothesized that experiencing greater friend support would predict trajectories and occasion-specific fluctuations characterized by lower levels of masculine-typed behavior. In all analyses, the potential moderating role of gender to account for differences in how these processes might play out for boys and girls was examined. Additionally, given potential racial/ethnic differences in how adolescents experience masculinity norms, our examination of trajectories of masculine-typed behaviors accounted for adolescents’ race/ethnicity and social class.

## Methods

### Participants

The sample for the present study comprised 334 adolescents from Project AHEAD, a national longitudinal study of adolescent development in the United States. The sample was almost evenly divided between girls (*n* = 164) and boys (*n* = 169), with one participant identifying as gender queer (removed from the analysis to avoid conflation with cisgender youth’ experiences). Regarding ethnicity/race, 50% of the sample was non-Hispanic White (*n* = 169), while 19% were African American (*n* = 65), 15% were Latino/a/x (*n* = 51), 9% were multiracial (*n* = 31), 4% were Asian American (*n* = 12), 1% identified as another ethnicity (*n* = 5), and >1% were Native American (*n* = 1). Mothers’ level of formal education was used as a proxy for social class (Harding, 2006), with 22% of the sample having mothers with a high school education or less (*n* = 77), 46% with some college (*n* = 159), and 31% with a 4-year degree or higher (*n* = 105).

In October 2019, parents of adolescents were recruited using a third-party research service, Bovitz®, which retains a nationally representative panel of research participants. A stratified random sample of this panel was drawn using national quotas for gender, racial/ethnic identity, parent education, and geographic region. Inclusion criteria were that adolescents had to be between 14 and 17 years of age, and be in the 9^th^, 10^th^, or 11^th^ grades at their schools. Just under 1,000 parents were contacted through the service’s online survey platform. A description of the study was provided that allowed parents to consent to their children’s participation. Parents were then asked to provide the survey to their adolescent child. In total, 570 adolescents assented and completed the survey at Time 1 (T1) in October 2019. Follow-up surveys were administered every six months thereafter (April 2020, October 2020, April 2021), for a total of four waves. At these ensuing waves, an email invitation was sent to all participants that included a link to the survey. Upon opening the invitation, parental consent and adolescent assent were obtained. Assenting adolescents were directed to the survey, which asked about their experiences and attitudes with academics, interpersonal relationships, and mental health. To ensure validity of responses, attention checks were implemented at each wave and all responses were back validated with prior waves to ensure consistency of identifying data (e.g., birthdates). Surveys took approximately 30 minutes to complete, and adolescents were compensated with a $20 Amazon e-gift card at each wave for participating. All procedures were approved by the Brigham Young University IRB.

A total of 570 adolescents began the study at Wave 1. For the purposes of this analysis, only those who participated in at least three of the four waves, for a total of 334 (59%). T-tests and chi-square analyses were conducted to examine patterns of attrition. Those who dropped out of the study were not meaningfully different than those who participated in 3 or more waves on most of the study variables, including the socio-demographic controls. The only exception was with homophobic victimization. Those who did not participate in at least 3 assessments reported slightly higher levels of homophobic victimization (*M* = 1.28, *SD* = 0.61) compared to those who were retained for 3 waves or more (*M* = 1.20, *SD* = 0.52). This may have been due to increased participant stress due to frequent victimization. However, this difference produced a minimal effect size (Cohen’s *d* = 0.15). Conventional interpretations of effect size consider anything lower than *d* = 0.20 to be negligible in practice (i.e., “merely statistical”; Fritz et al., 2012), so the analyses were retained as designed. Power analysis showed that a sample size of 300 is adequate to detect relatively small effects that are common in related research (*r* = 0.20; Faul et al., 2007). In multilevel models, the size of the highest-order level (in this case individuals) is the most important limiting factor of a study’s power (Snijders, 2005).

### Measures

#### Masculine-Typed Behaviors

At all four waves, masculine-typed behaviors were measured using the Adolescent Masculinity in Relationships Scale (Chu et al., 2005) as adapted by Rogers and colleagues (2017) to reflect the degree of endorsement of broadly recognized masculine gender roles within one’s relationships, including emotionally restrictive behavior, physical toughness and aggression. Specifically, participants indicated their level of agreement to eleven items on a Likert scale of 1 (*strongly disagree*) to 5 (*strongly agree*). Items were averaged such that higher scores indicated more traditionally masculine social behaviors. Example items include “I cannot respect a friend who backs down from a fight” and “If I tell my friends my worries, I will look weak.” See Appendix A for a complete list of items included in this measure. This scale demonstrated adequate internal consistency at all waves (W1 a = 0.82; W2 a = 0.82; W3 a = 0.84; W4 a = 0.84), and in prior studies has shown construct validity as a unidimensional assessment of endorsement of traditionally masculine behavior (Rogers et al., 2017).

#### Social Support

At all waves, participants reported their perceived degree of social support from friends using the friend’s subscale of the Multidimensional Scale of Perceived Social Support (Zimet et al., 1988). Participants indicated their agreement with four items such as *“I can count on my friends when things go wrong”* on a Likert scale of 1 (*strongly disagree*) to 7 (*strongly agree*). Responses were averaged such that higher scores reflected greater perceived social support (W1 a = 0.91; W2 a = 0.88; W3 a = 0.90; W4 a = 0.90).

#### Negative Peer Treatment

Negative peer treatment was assessed at all timepoints using four items from the Peer Interactions subscale of the Early Adolescent Role Strain Inventory (EARSI; Fenzel, 1989). Participants rated how often they experienced negative treatment by peers (e.g., “How often are other students mean to you?” and “How often do other students exclude you from activities?”) on a 5-point rating scale (1 = *Never*, 5 = *Almost Always)*. Items were averaged to create mean scores (α = 0.85), with higher scores reflecting more experiences of negative treatment from peers. Construct and convergent validity for the EARSI and its subscales has been demonstrated previously (Fenzel, 1989) and reliability was good at all waves in the present sample (W1 a = 0.86; W2 a = 0.85; W3 a = 0.87; W4 a = 0.87).

#### Homophobic Name-calling

Homophobic name-calling was measured at all waves using a modified version of the Homophobic Content Target Scale (Poteat & Espelage, 2005). The five-item scale was assessed using a 5-point Likert scale (*1=never, 2* = *1 or 2 times, 3* = *3 or 4 times, 4* = *5 or 6 times, 5* = *7 or more times*). Five items asked how often participants had been the victim of homophobic name-calling. An example item includes “How many times in the past week has a classmate called you [gay, lesbo, fag, etc.]?” Reliability for this measure was good (W1 a = 0.87; W2 a = 0.86; W3 a = 0.77; W4 a = 0.87).

#### Socio-demographic Variables

At wave 1, participants reported their gender (*0* = *girl, 1* = *boy*). They also reported their ethnic identity (African American, Asian American, Latinx/Hispanic, White, and Other). For analysis, dummy variables for individual ethnic groups were considered, but cell sizes for most minority groups were small and underpowered in later analyses. To avoid Type II error, ethnicity was recoded for *ethnic minority status* (*0* = *non-Hispanic white; 1* = *non-white ethnic minority*). Finally, they reported their mothers’ highest level of formal education (*1* = *Less than High School, 2* = *High School or Equivalent, 3* = *Some College or Vocational Degree, 4* = *Four-year College Degree, 5* = *Master’s Degree, 6* = *Doctoral or Professional Degree)*.

### Plan of Analysis

Intraclass correlations (ICCs) were estimated to determine the proportion of variance in masculine-typed behavior at the within and between-person levels. A multilevel modeling framework was used to estimate developmental trajectories in masculine-typed behaviors, with time being indicated by adolescents’ age (calculated by the date of their survey minus their date of birth). Multilevel models adapt elegantly to nested observations to produce *within-person* estimates of social processes (i.e., intercepts, slopes, or rates of change). They also allow for the estimation of individual differences in these within-person trends. Furthermore, in accounting for the nested nature of the data, between-person traits and characteristics are controlled by virtue of the design itself, further enabling the estimation of unique, time-specific intraindividual associations.

Primary analysis began by using a model building approach to first find the best fitting growth trajectories. First, a no-growth model centered at age 14, where the intercept (but no slope) was estimated. Following, a linear slope was introduced, indicated by adolescent age. Then, a quadratic term (age^2^) was added to the model. For example, the multilevel equation for a model retaining the linear slope would be expressed as:

Level 1 Model:

$${{{\mathrm{Masc}}}}_{{{{\mathrm{ij}}}}} = \beta _{0{{{\mathrm{i}}}}} + \beta _{1{{{\mathrm{i}}}}}\left( {{{{\mathrm{age}}}}} \right) + \varepsilon _{{{{\mathrm{ij}}}}}$$

Level 2 Model:

$$\beta _{0{{{\mathrm{i}}}}} = \gamma _0 + {{{\mathrm{U}}}}_0$$

$$\beta _{{{{\mathrm{ii}}}}} = \gamma _0 + {{{\mathrm{U}}}}_0$$

Interpreted, the masculinity score of an adolescent (i) at timepoint (j) was modeled at Level 1 as a function of an intercept, *β*_0_ (his/her cross-time average), a slope, *β*_1_ (the effect of his/her age), and residual within-person variance, ε. The intercept and slope were then modeled at Level 2 as a function of the sample average (γ_00 and_ γ_10_, respectively), and residual between-person variance (*U*_0i_ and *U*_1i_, respectively).

With the addition of each time polynomial (no-growth, linear slope, quadratic term), model fit was assessed using the -2 log likelihood (-2LL), the Akaike information criterion (AIC), the Bayesian information criterion (BIC) and the adjusted Bayesian information criterion (A-BIC). As these are comparative fit indices with no inherent metric or scaling, they are only useful for comparing increasingly complex, nested models (Field & Wright, 2011). Lower values indicated better fit to the data. A model was retained if it showed better fit than the previous, more parsimonious model.

### Predictors of Between- and Within-Person Variance in Masculine-typed Behavior

After fitting the most appropriate growth model, individual differences (between-person variance) were next examined in trajectories of masculine-typed behaviors. Adolescent sex, ethnic/racial minority status, and mother’s formal education (an indicator of social class; Kim et al., 2013) were included as time-invariant predictors of both the intercept and slope at Level 2. This model was expressed as:

Level 1 Model:

$${{{\mathrm{Masc}}}}_{{{{\mathrm{ij}}}}} = \beta _{0{{{\mathrm{i}}}}} + \beta _{1{{{\mathrm{i}}}}}\left( {{{{\mathrm{age}}}}} \right) + \varepsilon _{{{{\mathrm{ij}}}}}$$

Level 2 Model:

$$\begin{array}{l}\beta _{0{{{\mathrm{i}}}}} = \gamma _{00} + \gamma _{01}\left( {{{{\mathrm{sex}}}}} \right) + \gamma _{02}\left( {{{{\mathrm{minority}}}}\,{{{\mathrm{status}}}}} \right) \\\qquad+\, \gamma _{03}\left( {{{{\mathrm{mother}}}}\,{{{\mathrm{education}}}}} \right){{{\mathrm{ + U}}}}_{0{{{\mathrm{i}}}}}\end{array}$$

$$\begin{array}{l}\beta _{1{{{\mathrm{i}}}}} = \gamma _{10} + \gamma _{11}\left( {{{{\mathrm{sex}}}}} \right) + \gamma _{12}\left( {{{{\mathrm{minority}}}}\,{{{\mathrm{status}}}}} \right)\\ \qquad +\, \gamma _{13}\left( {{{{\mathrm{mother}}}}\,{{{\mathrm{education}}}}} \right){{{\mathrm{ + U}}}}_{1{{{\mathrm{i}}}}}\end{array}$$

Interpreted, the Level 2 equation now specified adolescents’ intercepts and slopes as a function of the sample average (γ_00_ and γ_10_ respectively); their gender (γ_01_ and γ_11_); their ethnic/racial minority status (γ_02_ and γ_12_); their social class (γ_03_ and γ_13_); and residual between-person variance (*U*_0*i* AND_ *U*_1*i*_). That is, variability in the intercepts and slopes of adolescents’ masculinity over time were predicted by gender, ethnic/racial minority status, and social class.

In a final step, peer interaction variables were entered as predictors of cross-time trajectories and occasion-specific fluctuations in masculinity. Specifically, friend support, negative peer treatment, and homophobic name-calling were included as Level 1, time-varying predictors of masculinity. Then, the cross-time averages of these same variables were included as Level 2 predictors of the intercept and slope.

Level 1 Model:

$$\begin{array}{ll}{{{\mathrm{Masc}}}}_{{{{\mathrm{ij}}}}} = \beta _{0{{{\mathrm{i}}}}} + \beta _{1{{{\mathrm{i}}}}}\left( {{{{\mathrm{age}}}}} \right) + \beta _{2{{{\mathrm{i}}}}}\left( {{{{\mathrm{friend}}}}\,{{{\mathrm{sup}}}}.} \right) \\\qquad\qquad+\, \beta _{3{{{\mathrm{i}}}}}\left( {{{{\mathrm{negative}}}}\,{{{\mathrm{treat}}}}.} \right) + \beta _{1{{{\mathrm{i}}}}}\left( {{{{\mathrm{homophobic}}}}\,{{{\mathrm{vict}}}}.} \right) + \varepsilon _{{{{\mathrm{ij}}}}}\end{array}$$

Level 2 Model:

$$\begin{array}{l}\beta _{0{{{\mathrm{i}}}}} = \gamma _{00} + \gamma _{01}\left( {{{{\mathrm{sex}}}}} \right) + \gamma _{02}\left( {{{{\mathrm{minority}}}}\,{{{\mathrm{status}}}}} \right)\\\qquad +\, \gamma _{03}\left( {{{{\mathrm{social}}}}\,{{{\mathrm{class}}}}} \right) + \gamma _{04}(\overline {friend\,{\it{sup}}} ) + \gamma _{05}(\overline {negative\,treat} )\\\qquad +\, \gamma _{06}(\overline {{\it{hom}}ophobic\,vict} ) + U_{0{{{\mathrm{i}}}}}\end{array}$$

$$\begin{array}{l}\beta _{1{{{\mathrm{i}}}}} = \gamma _{10} + \gamma _{11}\left( {{{{\mathrm{sex}}}}} \right) + \gamma _{12}\left( {{{{\mathrm{minority}}}}\,{{{\mathrm{status}}}}} \right)\\ \qquad+\, \gamma _{13}\left( {{{{\mathrm{social}}}}\,{{{\mathrm{class}}}}} \right) + \gamma _{14}(\overline {friend\,{\it{sup}}} ) + \gamma _{15}(\overline {negative\,treat} )\\\qquad\, + \gamma _{16}(\overline {{\it{hom}}ophobic\,vict} ) + U_{{{{\mathrm{0i}}}}}\end{array}$$

This model built on the models in the prior steps, such that the Level 1 equation now specified the masculinity score of an adolescent (i) at a specific timepoint (j) as a function of their intercept or cross-time average (*β*_0_), a slope or effect of their age (*β*_1_), their occasion-specific reports of friend support (*β*_2_), negative peer treatment (*β*_3_), homophobic name-calling (*β*_4_), and a within-person residual, ε. Then, at Level 2 the intercept and slope were each expressed as a function of a cross-time average (γ_00_ and γ_10_, respectively), the adolescents’ sex (γ_01;_ γ_11_), ethnic/racial minority status (γ_02;_ γ_12_), social class (γ_03;_ γ_13_), and their own cross-time averages of friend support (γ_04;_ γ_14_), negative peer treatment (γ_05;_ γ_15_) and homophobic name-calling (γ_06;_ γ_16_). For example, variability in a participant’s masculinity at a specific wave was predicted to fluctuate alongside their peer experiences; friend support, negative peer treatment, and homophobic name-calling at that same wave (Level 1). In addition, individual differences in masculinity trajectories across time were predicted by individual differences in the cross-time averages of these same peer experiences (Level 2).

To assist in interpretation of the resulting coefficients, the Level 1 predictors were group-mean centered, and the Level 2 predictors were grand-mean centered. For example, a significant effect of negative peer treatment at level 1 would indicate that on occasions in which adolescents experienced more negative peer treatment than their typical, cross-time average, they reported more elevated levels of masculine-typed behavior at that same time point. A significant effect of negative peer treatment at level 2 would indicate a contextual effect, such that adolescents with higher cross-time averages of negative peer treatment report a higher intercept or slope in masculinity, relative to the rest of the sample.

Altogether, this approach disaggregated time-specific and cross-time effects of peer interactions on masculinity scores. Specifically, it allowed for the estimation of (a) average within-person trajectories of masculine-typed behaviors from ages 14–17, (b) individual differences in these trajectories based on gender, ethnic/racial minority status, and social class, and finally (c) whether peer interactions could predict both cross-time trajectories in masculine-typed behavior, as well as time-specific fluctuations in the same. As a follow-up step, gender was included as a Level 2 moderator of these processes to examine if peer interactions were associated with masculinity differently for boys and girls. Analyses were conducted in Mplus v8.5 using full information maximum likelihood to handle cases with missing data (FIML; Enders, 2022).

## Results

### Descriptive Statistics

Variable means and standard deviations are presented in Table 1. At all waves, boys scored higher on masculine-typed behavior than girls. However, both boys and girls remained below the midpoint of the scale, reflecting low to moderate overall levels of masculine-typed behavior. Girls and boys reported moderate-to-high levels of friend support, and girls scored higher on this measure at all waves with a small effect size. Adolescents reported low average levels of negative peer treatment and homophobic victimization. Bivariate correlations are presented in Table 2. Masculine-typed behavior was negatively associated with friend support at all waves, and positively associated with and negative peer treatment and homophobic name-calling at all waves, although the latter relations were weaker.

**Table 1:** Means and standard deviations of continuous study variables at Time 1

Variable	Boys		Girls		
	*M*	*SD*	*M*	*SD*	*d*
Masculine-typed Behavior (T1)	2.79	0.61	2.25	0.59	0.89
Masculine-typed Behavior (T2)	2.71	0.60	2.23	0.59	0.74
Masculine-typed Behavior (T3)	2.70	0.61	2.19	0.62	0.73
Masculine-typed Behavior (T4)	2.65	0.60	2.17	0.65	0.73
Friend Support (T1)	5.41	1.18	5.79	1.01	-0.35
Friend Support (T2)	5.41	1.10	5.82	0.96	-0.40
Friend Support (T3)	5.47	1.12	5.80	1.09	-0.30
Friend Support (T4)	5.57	0.93	5.77	1.11	-0.18
Negative Peer Treatment (T1)	1.67	0.77	1.60	0.71	*ns*
Negative Peer Treatment (T1)	1.61	0.69	1.58	0.72	*ns*
Negative Peer Treatment (T1)	1.43	0.66	1.48	0.60	*ns*
Negative Peer Treatment (T1)	1.48	0.64	1.49	0.72	*ns*
Homophobic Victimization (T1)	1.28	0.63	1.11	0.36	0.12
Homophobic Victimization (T1)	1.23	0.47	1.15	0.45	*ns*
Homophobic Victimization (T1)	1.23	0.50	1.15	0.36	*ns*
Homophobic Victimization (T1)	1.29	0.59	1.25	0.57	*ns*

Cohen’s *d* values are presented for those mean differences that are statistically significant between boys and girls. Positive *d*-scores represent differences in which boys scored higher, negative *d*-scores represent differences in which girls scored higher

**Table 2:** Bivariate correlations

		*1*	*2*	*3*	*4*	*5*	*6*	*7*	*8*	*9*	*10*	*11*	*12*	*13*	*14*	*15*
*1*	Masculine-typed behavior W1	–														
*2*	Masculine-typed behavior W2	0.75***	–													
*3*	Masculine-typed behavior W3	0.67***	0.76***	–												
*4*	Masculine-typed behavior W4	0.60***	0.63***	0.70***	–											
*5*	Social Support- Friends W1	–0.42***	–0.35***	–0.34***	–0.23***	–										
*6*	Social Support- Friends W2	–0.39***	–0.44***	–0.41***	–0.34***	0.51***	–									
*7*	Social Support- Friends W3	–0.30***	–0.38***	–0.43***	–0.34***	0.62***	0.57***	–								
*8*	Social Support- Friends W4	–0.36***	–0.44***	–0.43***	–0.34***	0.56***	0.60***	0.65***	–							
*9*	Negative Peer Treatment W1	0.23***	0.27***	0.27***	0.28***	–0.20***	–0.28***	–0.18**	–0.21***	–						
*10*	Negative Peer Treatment W2	0.14*	0.26***	0.24***	0.25***	–0.14*	–0.23***	–0.13*	–0.23***	0.63***	–					
*11*	Negative Peer Treatment W3	0.07	0.14*	0.18**	0.17**	–0.15**	–0.15**	–0.27***	–0.21***	0.45***	0.54***	–				
*12*	Negative Peer Treatment W4	0.17	0.21***	0.21**	0.36***	–0.14*	–0.20**	–0.19**	–0.22***	0.59***	0.67***	0.58***	–			
*13*	Homophobic Name-calling W1	0.21***	0.11*	0.13*	0.19**	–0.02	–0.13*	–0.03	–0.03	0.14**	0.08	0.02	0.26***	–		
*14*	Homophobic Name-calling W2	0.20***	0.19***	0.18**	0.28***	0.04	−0.07	−0.01	–0.01	0.16**	0.24***	0.002	0.31***	0.36***	–	
*15*	Homophobic Name-calling W3	0.13*	0.13*	0.11*	0.15*	−0.01	−0.12*	−0.08	–0.05	0.28***	0.22***	0.30***	0.30***	0.30***	0.32***	–
*16*	Homophobic Name-calling W4	0.18**	0.25***	0.23**	0.40***	−0.07	−0.05	−0.09	–0.05	0.28***	0.31***	0.25***	0.47***	0.29***	0.39***	0.31***

*p < 0.05; **p < 0.01; ***p < 0.001

### Assessing Sources of Variance

ICCs were calculated for each of the observed repeated measures of masculinity. Because the grouping variable in the present application is the individual, an ICC represents the total variance in a repeated measure attributable to individual, trait-like differences (i.e., between-person variance). The ICC for adolescents’ masculinity scores was ICC = 0.70. Interpreted, 70% of all the variance in adolescents’ masculine-typed behavior was attributable to individual (trait-like) differences between participants. However, this also means that substantial variance – upwards of 30% - represented time-specific, within-person fluctuations (state-like) in which adolescents deviated from their own cross-time averages. This degree of within-person variance justifies the ensuing analysis of how masculinity changes within and across time, as well as an exploration of the factors that predicts these time-specific fluctuations.

### Change in Masculine-typed behaviors from Age 14 to 17

Table 3 presents the fit indices for the model building process in which a best-fitting growth solution was explored. The linear model showed better fit to the data than the unconditional, no-growth model. However, the quadratic model did not improve model fit over the linear model. As such, the linear model was retained as the best-fitting model to describe overall change patterns in masculine-typed behaviors across the sample. Table 3 presents the parameter estimates of this linear growth model, which was centered at age 14. The results showed that adolescents reported moderate-to-low initial levels of masculine-typed behaviors at age 14. The linear slope was negative and statistically significant, indicating that adolescents showed an average linear decline in masculine-typed behaviors through age 17.

**Table 3:** Growth models fit indices

	AIC	BIC	A-BIC
No growth	1723.319	1738.705	1729.176
**Linear**	**1688.222**	**1718.872**	**1699.813**
Quadratic	1682.335	1733.417	1701.653

Bolded row indicates the optimal fitting solution

Next, the effect of between-person (time-invariant) background characteristics were used as predictors of the intercept and slope in masculine-typed behavior. Results are presented in Table 4. The intercept was positively associated with adolescent gender, indicating that boys reported higher levels of masculine-typed behavior at age 14 than girls. Neither ethnic/racial minority status nor social class were associated with the intercept, meaning that adolescents of varying racial/ethnic identities and social classes showed similar levels of masculine-typed behavior at age 14. Similarly, none of the demographic background variables were associated with the slope. This included adolescent gender, meaning that boys and girls showed similar rates of decline in masculinity over time. Thus, boys reported higher levels of masculinity than girls at age 14, a difference that was sustained through age 17 given their similar rates of decline.

**Table 4:** Parameter estimates for growth model and then models with predictors

	Fixed parameter estimates		
	Linear Model	Linear Model: Demographics	Linear Model: Peer Interact.
Level 1 Prediction			
Intercept	2.59***	2.34***	2.38***
Slope	−0.04**	−0.04*	0.02
Friend Support			−0.07*
Negative Peer Treatment			0.03
Homophobic Victimization			0.09***
Level 2 Prediction			
Prediction of Intercept			
Gender		0.58***	0.38***
Minority Status		0.07	0.03
Parent Education		−0.20	0.09
Friend Support			−0.24***
Negative Peer Treatment			0.08
Homophobic Victimization			0.33**
Prediction of Slope			
Gender		−0.02	−0.01
Minority Status		0.01	0.01
Parent Education		−0.01	0.01
Friend Support			−0.01
Negative Peer Treatment			0.01
Homophobic Victimization			−0.03

Growth models all centered at age 14. Within-person predictors were cluster-mean centered; between person predictors were grand-mean centered

*p < 0.05, **p < 0.01, ***p < 0.001

### Peer Experiences and Occasion-Specific Fluctuations in Masculinity

Next, peer interactions were entered as predictors of cross-time trajectories of masculine-typed behavior from age 14 to 17 (Level 2), as well as time specific fluctuations in masculine-typed behavior (Level 1). The results are presented in Table 4. In the Level 2 equation, friend support was negatively associated with the intercept, and homophobic name-calling was positively associated with the intercept. Negative peer treatment was unassociated with the intercept. None of the peer interaction variables were associated with the slope. Together, the emergent pattern was that adolescents who reported higher cross-time averages of social support reported lower initial levels of masculine-typed behavior at age 14, relative to others. Adolescents experiencing higher cross-time averages of homophobic name-calling reported higher initial levels of masculinity at age 14, relative to others. As these variables were unassociated with the slope, these individual differences were sustained through age 17.

In the Level 1 equation, the effect of friend support was negative and significant, while the effect of negative peer treatment was positive and significant. On occasions when adolescents experienced more friend support than their own cross-time trajectories, they reported lower levels of masculine-typed behavior. On occasions when adolescents saw higher negative treatment than their own cross-time trajectories, they reported higher levels of masculine-typed behavior. There were no associations with homophobic name-calling in the Level 1 equation.

As a sensitivity analysis, adolescent gender was examined as a possible moderator for the effects of peer experiences, entering gender as a predictor of the Level 1 effects in a Level 2 interaction. None of these interaction terms were significant (social support, β = 0.05, SE = 0.04, *p* = 0.21; negative treatment; β = −0.02, SE = 0.07, *p* = 0.74; homophobic name-calling; β = −0.07, SE = 0.07, *p* = 0.30). Thus, while the overall trajectories showed higher levels of masculine-typed behavior for boys across time compared to girls, the level-1 time-specific effects of social support, negative peer treatment and homophobic name-calling on masculine-typed behavior were similar for boys and girls.

## Discussion

Little is known about how masculine-typed behavior within friendships develops over time during middle adolescence – particularly in the context of peer experiences for boys and girls. To fill this gap, this study examined trajectories of adolescents’ masculine-typed behaviors from ages 14 to 17, while also accounting for time-specific fluctuations in these behaviors. How changes in masculine-typed behavior were associated with peer interactions, including friend support, negative peer treatment, and homophobic victimization was also identified.

### Understanding Change in Masculine-typed Behaviors in Middle Adolescence

The results showed that boys and girls alike showed overall decreases in their adherence to masculine-typed behaviors from age 14 to 17. Boys began middle adolescence showing greater adherence to masculine-typed behaviors compared to girls, but both boys and girls started below the midpoint on the scale of masculine-typed behavior, and then showed similar rates of decline as they aged. This may seem surprising given the high cultural value and salience of traditional masculinity in Western societies (e.g., Duckworth & Trautner, 2019), but it is nevertheless consistent with many studies showing a considerable degree of resistance to masculinity among adolescents, including boys. For example, most studies on adolescent masculinity show boys and girls typically express disagreement with masculine bravado as a standard of behavior. Qualitative studies demonstrate the complexity with which boys navigate masculinity, with many boys actively resisting these norms to some degree (Way, 2011; Way et al. 2014). From a feminist lens, the observed decrease in masculine-typed behaviors in the present study may reflect adolescents’ sustained attempts to resist and renegotiate masculine norms as they grow. Of course, it is important to bear in mind that prior studies have shown boys to increase in masculine-typed behaviors during early adolescence. Early adolescence is a uniquely unstable period regarding peer networks, and so the calculus in weighing the costs and benefits of masculine bravado may be different during this period. In early adolescence, masculine-typed behaviors may have more utility in finding status within a peer group when social ties are few. However, as adolescents move into middle adolescence and beyond, peer networks tend to stabilize, and this may change the cost-benefit analysis of enacting masculine-typed behaviors. Thus, although masculine behaviors are one pathway to peer belonging in the broader group (Way, 2013) – our analyses suggest that adolescents may grow increasingly resistant to masculine-typed behaviors. As our measure of masculine-typed behaviors was focused on these behaviors within the context of friend-level interactions, this is a plausible interpretation, as it is still likely that these same adolescents may still engage in masculine-typed behaviors when interacting with the larger peer network. In this way, our findings dovetail well with recent research and news media highlighting men and boys attempts to redefine masculinity (e.g., Bogen et al., 2021) – especially within the context of close relationships such as friendships. That these trends of resistance held across gender, race/ethnicity, and social class may further indicate the widespread scope of resistance among adolescents in the United States towards masculine-typed behaviors within the context of friendships.

Of course, the trend toward declining masculine-typed behaviors was an average trend that unfolded across time. A key additional insight from the results was that individual adolescents also experienced meaningful fluctuations in masculine-typed behavior at each wave. In other words, although adolescents showed an overall trend toward decline in masculinity, there were still deviations around those overall downward trends. The negotiation of gender roles, including with masculinity, involves balancing gender-role conformity and gender-role resistance (Chu, 2014), not just across time but also in response to situational demands. This likely evidences the contextual salience of masculinity and justifies continuing research on contextual factors that may raise or diminish the salience of traditional masculinity for adolescents. Furthermore, the lack of significant predictors of the slope of masculinity points perhaps to a developmental trend in accommodation to masculine-type behaviors. This may be due to the possibility of increased stability of adolescent peer groups during the latter part of adolescence, or other more generalized experiences such as socialization via social media. Future research, however, would do well to further explore this finding to see if it replicates in other samples or if other peer experiences shape the overall trajectory of masculine-typed behaviors around friends.

### Peer Experiences and Occasion-Specific Fluctuations in Masculinity

This study also examined peer experiences as a salient context that may drive cross-time and time-specific changes in masculine-typed behaviors. Consistent with hypotheses, our findings provided compelling evidence that peer interactions are a key correlate of masculine-typed behavior. At both the between- and within-person levels, feeling more supported by friends was associated with less masculine-typed behavior. Adolescents reporting higher friend support reported fewer masculine typed behaviors at the outset of the study, and on occasions in which adolescents experienced more friend support than their cross-time trajectory, they also reported lower levels of masculine-typed behaviors than typical. In short, the least masculine-typed behavior was reported by adolescents who felt connected to and supported by friends. The converse is also true: the most masculine-typed behavior was reported by adolescents experiencing marginalizing peer interactions, including homophobic victimization and peer harassment. For example, at the between-person level, adolescents who reported higher homophobic victimization reported more masculine-typed behaviors. At the within-person level, on occasions in which adolescents experienced more negative peer treatment than typical, they also reported more masculine-typed behavior than typical.

Taken together, the findings seem to reflect the social paradox of traditional masculinity for adolescents: although masculine-typed behaviors are often a criterion for belonging and acceptance in broader peer groups – particularly for boys – they are also antithetical to the maintenance close friendships (Gupta et al., 2013). It is possible that as intimacy needs are satisfied, masculine edicts for assertive and status-seeking behaviors among friends may seem less relevant, needful, and even viable. Indeed, prior studies have found that well-liked adolescents have more social leeway to resist traditional masculine norms (Way, 2011). Instead, masculine-typed behaviors for toughness and stoicism may be perceived to have more social utility among those adolescents who are victimized or marginalized, where it may be used as a coping response. Indeed, one might expect adolescents who display fewer masculine-typed behaviors to be targeted for increased homophobic victimization (the opposite of what our analyses suggest). However, our measure of masculine-typed behaviors focused on friendships (and not the larger peer network). This nuance suggests that when adolescents are more often called homophobic slurs, they feel the need to broadcast more masculine-type behaviors among their friends – likely to maintain their standing within their friendships. Furthermore, familiar masculine-typed behaviors may serve as a coping response in response to peer victimization, as the individual responds to a social stressor by aggressing, and/or not displaying vulnerable emotions, like sadness or fear.

Of course, the associations between interpersonal relationships and masculinity could proceed in the other direction. The findings at the within-person level (time-specific) were technically concurrent associations, not lagged. Therefore, it is also possible that adolescents who display lower levels of masculine-typed behaviors invite greater levels of friend support and lower levels of negative treatment. Adolescents who are less traditionally masculine may feel increased permission to develop meaningful friendships which provide them with increased support. Alternatively, adolescents who are highly masculine may struggle in developing close friendships, which can leave them vulnerable to continued negative treatment among peers. As all these explanations remain theoretically viable, future research might explore the directionality in interpersonal relationships and masculine-typed behavior over time. Furthermore, as our analyses indicate that overall adherence to masculinity decreases with time, this could mean that adolescents who are continuing to broadcast elevated levels of masculine-typed behaviors may continue to experience greater feelings of marginalization from their peer group as masculine-typed behaviors becomes less and less valued and necessary to fit into the peer group. Irrespective of the directionality of these processes however, for the present purposes, our findings show support for feminist and developmental-contextual suppositions that masculinity is inherently relational in nature (Chu, 2014), meaning that it is in the context of social and interpersonal relationships that masculine norms find value and meaning for adolescents, and therefore application.

### Implications

The present findings have important implications for research and practice. When considering the importance of peer groups during adolescence (Hamm & Faircloth, 2005) for educational engagement and achievement (Wang et al., 2018), mental health in adulthood (Narr et al., 2017), and the development of problematic behaviors (e.g., Savolainen et al., 2018), it is imperative to acknowledge the ways in which the nuances around adherence to masculine-typed behaviors may make it increasingly difficult for adolescents to find belonging. To this end, our findings may provide some useful guidance for prevention and intervention, particularly those that aim to counter harmful gender stereotypes surrounding masculinity. For example, our research indicates that boys and girls in adolescence are increasingly resistant to traditional approaches to masculinity – at least within the context of friendships. This may cut against many current perspectives in scholarship and in popular media, for example those that raise alarm about “boy crises” (e.g., Sax, 2007). While there are negative implications of traditionally masculine-typed behaviors for development, there is value gained in recognizing that there is opposition to many of these negative depictions among boys and girls, which may be leverageable in prevention and intervention efforts. This could involve helping adolescents deconstruct masculine-typed behaviors and challenge its place as the “right” approach to being male, for example, while also presenting positive viable alternatives (see Bogen et al., 2021).

Building on this point, and within these same efforts, our findings point to the value of leveraging social and interpersonal relationships to further shape resistance to traditionally masculine norms. Interventions that promote meaningful connection among adolescents and which decrease broader peer victimization may benefit them in regard to how they navigate salient gender norms. Within this same context, our findings may provide information to help identify at-risk adolescents. Strong adherence to masculine-typed behaviors may be an indicator of negative social interactions, specifically negative treatment from peers. Attempts to help adolescents resist masculine-typed behaviors may be less effective if adolescents do not feel they possess the social capital to risk deviating from the strong and stoic that traditional masculinity presents. As such, helping adolescents to build meaningful social relationships is perhaps an effective means by which parents and educators can help adolescents resist masculine-typed behaviors, and the negative processes and outcomes linked to these behaviors ranging from mental health challenges (Rogers et al., 2017) to substance abuse (Fugitt & Ham, 2018).

### Limitations and Future Research

While this research has the strength of a multilevel longitudinal design, there are also several limitations. First, although the adolescents in our sample come from diverse racial/ethnic backgrounds, the cell sizes for many of the ethnic-minority groups were small and underpowered, constraining our ability to understand how BIPOC adolescents must uniquely navigate masculine norms. As different racial and ethnic groups often approach and transmit ideals of masculinity differently (e.g., Silva, 2021), more focused analyses on BIPOC adolescents are necessary to truly understand how adolescence negotiate masculine norms within and across time. Future research should examine trajectories of masculine identity in larger samples of minority populations – including those outside of the United States. Similar comments can be made of gender-diverse adolescents (e.g., trans, non-binary), of whom the sample included only a few.

Finally, our analysis focused on adherence to masculine-typed behaviors, although our measure also included items which addressed internal feelings towards masculine-typed ideologies and behaviors. As there are many different aspects of masculinity, future research should examine trajectories of adherence to a wider scope of masculine norms (such as risk taking, self-reliance, being a playboy) as well as feminine norms, and within different contexts outside of adolescent’s friendships. Furthermore, future research would do well to examine the differences between enacted masculine behaviors and adolescents’ feelings towards different masculine norms. In addition, a deeper analysis on the peer context these behaviors take place in would be useful (e.g., friends vs. broader peer group), as our analyses suggest that masculine-typed behaviors are a result of peer experiences. To this point, when considering the specific context of peer groups in shaping masculine-typed behaviors and ideology, future research would do well to consider how peer experiences influence the normative group processes. This may be insightful, as it is possible that peer groups in which more positive experiences take place may also value masculinity less – while the inverse may be true for peer groups in which more negative experiences take place in. This research would provide a broader picture of how masculine identity holistically changes in a peer context across adolescence and help develop a better picture of what resistance to masculine-typed behaviors looks like in practice.

## Conclusion

To better understand how masculine-typed behaviors within friendships develops over time during middle adolescence – contextualized within peer experiences for boys and girls - this study examined (a) adolescents’ intra-individual trajectories of behavioral adherence to masculine-typed behaviors from ages 14 to 17, (b) individual differences in these trajectories according to sociodemographic background characteristics (e.g., sex, ethnic identity), and (c), and how peer-related experiences (homophobic name-calling and negative treatment) predicted within-person fluctuations and individual differences in masculinity during this time. Results indicate that adherence to masculine-typed behaviors decreased across later adolescence, regardless of gender and sociodemographic factors. Furthermore, adolescents who reported higher levels of negative treatment and homophobic name-calling reported higher adherence to masculine-typed behaviors. In contrast, adolescents who reported higher levels of peer support also reported lower adherence to masculine-typed behaviors. These patterns help us to better understand first, the development of masculine identity during adolescence, and second, how peer experiences relate to the development of masculine identity. Our hope is that this research will continue to aid a deeper understanding of how masculine identity develops, and how parents and educators can help adolescence approach the formation of their own masculine identity.

## References

[CR1] Bogen KW, Williams SL, Reidy DE, Orchowski LM (2021). We (want to) believe in the best of men: A qualitative analysis of reactions to #Gillette on Twitter. Psychology of Men & Masculinities.

[CR2] Brechwald WA, Prinstein MJ (2011). Beyond homophily: A decade of advances in understanding peer influence processes: beyond homophily. Journal of Research on Adolescence.

[CR3] Brown, L. M., & Gilligan, C. (1992). *Meeting at the crossroads: Women’s psychology and girl’s development*. Harvard University Press. 10.4159/harvard.9780674731837

[CR4] Buschmeyer A, Lengersdorf D (2016). The differentiation of masculinity as a challenge for the concept of hegemonic masculinity. NORMA.

[CR5] Chesney-Lind, K. I. M. (2011). Girls’ Violence: Beyond Dangerous Masculinity. In *Feminist Theories of Crime*. Routledge

[CR6] Chu JYC (2014). Supporting boys’ healthy resistance to masculine norms. Psychology of Men & Masculinity.

[CR7] Chu JY, Porche MV, Tolman DL (2005). The adolescent masculinity ideology in relationships scale: Development and validation of a new measure for boys. Men and Masculinities.

[CR8] Connell RW (2005). Growing up Masculine: Rethinking the Significance of Adolescence in the Making of Masculinities. Irish Journal of Sociology.

[CR9] Duckworth KD, Trautner MN (2019). Gender goals: Defining masculinity and navigating peer pressure to engage in sexual activity. Gender & Society.

[CR10] Enders, C. K. (2022). *Applied Missing Data Analysis, Second Edition (Methodology in the Social Sciences) (Second ed.)*. New York, NY: The Guilford Press.

[CR11] Faul F, Erdfelder E, Lang AG, Buchner A (2007). G* Power 3: A flexible statistical power analysis program for the social, behavioral, and biomedical sciences. Behavior research methods.

[CR12] Fenzel LM (1989). Role strain in early adolescence: A model for investigating school transition stress. The Journal of Early Adolescence.

[CR13] Field AP, Wright DB (2011). A primer on using multilevel models in clinical and experimental psychopathology research. Journal of Experimental Psychopathology.

[CR14] Fritz CO, Morris PE, Richler JJ (2012). Effect size estimates: current use, calculations, and interpretation. Journal of Experimental Psychology: General.

[CR15] Fugitt JL, Ham LS (2018). Beer for “brohood”: A laboratory simulation of masculinity confirmation through alcohol use behaviors in men. Psychology of Addictive Behaviors.

[CR16] Gupta T, Way N, McGill RK, Hughes D, Santos C, Jia Y, Yoshikawa H, Chen X, Deng H (2013). Gender-typed behaviors in friendships and well-being: A cross-cultural study of Chinese and American boys. Journal of Research on Adolescence.

[CR17] Hamm, J. V. & Faircloth, B. S. (2005). The role of friendship in adolescents’ sense of school belonging. *New Directions for Child and Adolescent Development*, (107), 61–78. 10.1002/cd.121. 10.1002/cd.121 15934221

[CR18] Harding NN (2006). Ethnic and social class similarities and differences in mothers’ beliefs about kindergarten preparation. Race Ethnicity and Education.

[CR19] Ioverno S, DeLay D, Martin CL, Hanish LD (2021). Who Engages in Gender Bullying? The Role of Homophobic Name-Calling, Gender Pressure, and Gender Conformity. Educational Researcher.

[CR20] Jackson C, Dempster S (2009). ‘I sat back on my computer … with a bottle of whisky next to me’: Constructing ‘cool’ masculinity through ‘effortless’ achievement in secondary and higher education. Journal of Gender Studies.

[CR21] Kim Y, Sherraden M, Clancy M (2013). Do mothers’ educational expectations differ by race and ethnicity, or socioeconomic status?. Economics of Education Review.

[CR22] Kleiser M, Mayeux L (2021). Popularity and gender prototypicality: An experimental approach. Journal of Youth and Adolescence.

[CR23] Leaper C, Farkas T, Starr CR (2019). Traditional masculinity, help avoidance, and intrinsic interest in relation to high school students’ English and math performance. Psychology of Men & Masculinities.

[CR24] Levant RF, Wimer DJ, Williams CM (2011). An evaluation of the Health Behavior Inventory-20 (HBI-20) and its relationships to masculinity and attitudes towards seeking psychological help among college men. Psychology of Men & Masculinity.

[CR25] Marcell AV, Eftim SE, Sonenstein FL, Pleck JH (2011). Associations of family and peer experiences with masculinity attitude trajectories at the individual and group level in adolescent and young adult males. Men and Masculinities.

[CR26] Martin-Storey A, August EG (2016). Harassment due to gender nonconformity mediates the association between sexual minority identity and depressive symptoms. Journal of Sex Research.

[CR27] Narr RK, Allen JP, Tan JS, Loeb EL (2017). Close friendship strength and broader peer group desirability as differential predictors of adult mental health. Child Development.

[CR28] Nielson MG, Rogers AA, Cook RE (2022). Nuanced longitudinal effects of domains of perceived gender similarity on adolescent peer victimization. Sex Roles.

[CR29] Pascoe, C. J. (2014). *Dude, you’re a fag: Masculinity and sexuality in high school* (2nd ed.). University of California Press; 10.1007/s13398-014-0173-7.2

[CR30] Perry DG, Pauletti RE, Cooper PJ (2019). Gender identity in childhood: A review of the literature. International Journal of Behavioral Development.

[CR31] Poteat VP, Espelage DL (2005). Exploring the relation between bullying and homophobic verbal content: The Homophobic Content Agent Target (HCAT) scale. Violence and Victims.

[CR32] Rapee RM, Magson NR, Forbes MK, Richardson CE, Johnco CJ, Oar EL, Fardouly J (2022). Risk for social anxiety in early adolescence: Longitudinal impact of pubertal development, appearance comparisons, and peer connections. Behaviour Research and Therapy.

[CR33] Reigeluth CS, Addis ME (2016). Adolescent boys’ experiences with policing of masculinity: Forms, functions, and consequences. Psychology of Men & Masculinity.

[CR34] Rogers AA, Cook RE, Guerrero K (2022). Is My Femininity a Liability? Longitudinal Associations between Girls’ Experiences of Gender Discrimination, Internalizing Symptoms, and Gender Identity. Journal of Youth and Adolescence.

[CR35] Rogers AA, DeLay D, Martin CL (2017). Traditional masculinity during the middle school transition: Associations with depressive symptoms and academic engagement. Journal of Youth and Adolescence.

[CR36] Rogers AA, Nielson MG, Santos CE (2021). Manning up while growing up: A developmental-contextual perspective on masculine gender-role socialization in adolescence. Psychology of Men & Masculinities.

[CR37] Roach A (2018). Supportive Peer Relationships and Mental Health in Adolescence: An Integrative Review. Issues in Mental Health Nursing.

[CR38] Rogers LO, Yang R, Way N, Weinberg SL, Bennet A (2019). “We’re supposed to look like girls, but act like boys”: Adolescent girls’ adherence to masculinity norms. Journal of Adolescence.

[CR39] Savolainen I, Sirola A, Kaakinen M, Oksanen A (2018). Peer group identification as determinant of youth behavior and the role of perceived social support in problem gambling. Journal of Gambling Studies.

[CR40] Sax, L. (2007). *Boys adrift: The five factors driving the growing epidemic of unmotivated boys and underachieving young men* (267). New York: Basic Books.

[CR41] Silva T (2021). Masculinity attitudes in the United States across intersections of race/ethnicity, immigration status, and education. Journal of Gender Studies.

[CR42] Snijders TA (2005). Power and sample size in multilevel modeling. In B.S. Everett and D.C. Howell (Eds.). Encyclopedia of statistics in behavioral science.

[CR43] Wang MT, Kiuru N, Degol JL, Salmela-Aro K (2018). Friends, academic achievement, and school engagement during adolescence: A social network approach to peer influence and selection effects. Learning and Instruction.

[CR44] Way N (2013). Boys’ Friendships During Adolescence: Intimacy, Desire, and Loss. Journal of Research on Adolescence.

[CR45] Way, N. (2011). *Deep secrets: Boys’ friendships and the crisis of connection*. Harvard University Press. 10.4159/harvard.9780674061361

[CR46] Way N, Cressen J, Bodian S, Preston J, Nelson J, Hughes D (2014). “It might be nice to be a girl… Then you wouldn’t have to be emotionless”: Boys’ resistance to norms of masculinity during adolescence. Psychology of Men & Masculinity.

[CR47] Wong YJ, Ho M-HR, Wang S-Y, Miller ISK (2017). Meta-analyses of the relationship between conformity to masculine norms and mental health-related outcomes. Journal of Counseling Psychology.

[CR48] Zimet GD, Dahlem NW, Zimet SG, Farley GK (1988). The multidimensional scale of perceived social support. Journal of Personality Assessment.

